# Activated protein C does not increase in the early phase of trauma with disseminated intravascular coagulation: comparison with acute coagulopathy of trauma-shock

**DOI:** 10.1186/s40560-015-0123-2

**Published:** 2016-01-04

**Authors:** Subrina Jesmin, Satoshi Gando, Takeshi Wada, Mineji Hayakawa, Atsushi Sawamura

**Affiliations:** Division of Acute and Critical Care Medicine, Department of Anesthesiology and Critical Care Medicine, Hokkaido University Graduate School of Medicine, N15W7, Kita-ku, Sapporo, 060-8638 Japan

**Keywords:** Trauma, Coagulopathy, Disseminated intravascular coagulation (DIC), Activated protein C

## Abstract

We hypothesized that activated protein C does not increase in disseminated intravascular coagulation (DIC) after trauma and that the same is true for acute coagulopathy of trauma-shock (ACOTS). Activated protein C levels were prospectively measured in 57 trauma patients: 30 with DIC and 27 without DIC. Normal to more decreased activated protein C levels were observed in DIC patients than in the controls and non-DIC patients. The activated protein C levels in ACOTS patients were similar to those in DIC patients. In conclusion, activated protein C does not increase in either DIC or ACOTS in the early phase of trauma.

## Findings

The present study demonstrated lower to normal activated protein C levels in DIC patients in the early phase of trauma. The same was true in ACOTS patients.

### Introduction

An impairment of the anticoagulation pathways, including protein C and thrombomodulin system, has been confirmed in disseminated intravascular coagulation (DIC), which suggests lower levels or dysfunction of activated protein C (APC), leading to systemic thrombin generation [1]. On the contrary, higher levels of APC and newly synthesized thrombomodulin with full function play pivotal roles in the development of thrombin shutoff in acute coagulopathy of trauma-shock (ACOTS) [2, 3]. The purpose of this study was to test the hypothesis that APC does not increase in DIC at the early phase of trauma and compare the obtained results with those in ACOTS.

### Methods and results

With the approval of the Institutional Review Board (No. 013–0215), 57 severe trauma patients defined as having an Injury Severity Score (ISS) ≥9 (at least one abbreviated injury scale ≥3) were enrolled. The patient population was same as our previous studies [4, 5]. Twelve healthy volunteers not matched by age served as control subjects. The diagnosis of DIC was made based on the Japanese Association for Acute Medicine (JAAM) DIC diagnosis criteria on the day of the admission. The overt DIC scores based on the International Society on Thrombosis and Haemostasis (ISTH) were also calculated. ACOTS was defined as a prothrombin time ratio >1.2. The APC levels were measured by an ELISA kit (USCN Life Science Inc. Wuhan; Cloud-Clone Corp., Houston, TX) within 12 h after arrival to the emergency department. The Mann-Whitney *U* test was performed to compare the APC levels and *p* values <0.05 were considered statistically significant. The IBM SPSS 22.0 for MAC OSX software program (IBM Japan, Tokyo) was used for the statistical analyses and calculations.

**Fig. 1:**
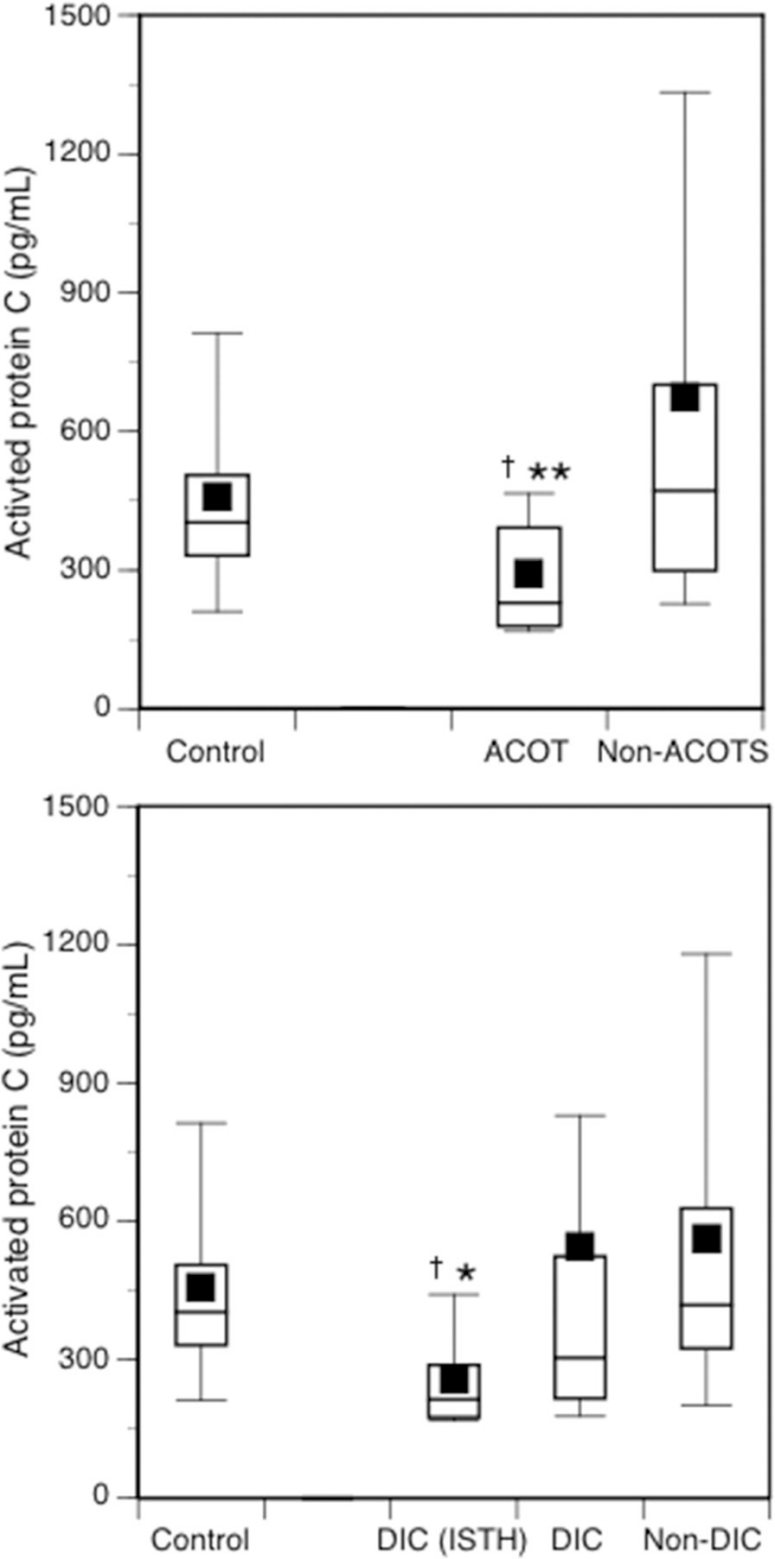
The APC levels in DIC (*bottom*) and in ACOTS (*top*). DIC (ISTH), those simultaneously met both JAAM DIC and ISTH overt DIC criteria; DIC, those met only JAAM DIC criteria; Non-DIC, they did not meet DIC criteria. The numbers of patients are as follows: control (*n* = 8), DIC (ISTH) (*n* = 9), DIC (*n* = 21), non-DIC (*n* = 27), ACOTS (*n* = 18), and non-ACOTS (*n* = 39). *Horizontal bars* in the box indicate the median (middle) and interquartile ranges (upper 25%, lower 75%). *Top and bottom bars* indicate the maximum and minimum values, respectively. Black squares are mean values. ^†^ *p* < 0.05 vs. control subjects; *p < 0.05, **p < 0.01 vs. non-DIC patients or non-ACOTS patients, respectively

The 57 patients were classified into 30 patients with DIC and 27 patients without DIC. Baseline characteristics (age, sex, and ISS) were identical between the two groups. Nine of 30 DIC patients simultaneously met the ISTH overt DIC criteria. Eighteen patients met the definition of ACOTS. There was no difference in the APC levels between the control and DIC patients. In patients who simultaneously met the ISTH over DIC criteria, however, significantly lower APC levels than those in the controls and non-DIC patients were observed. ACOTS patients showed significantly lower levels of APC than those in the controls and non-ACOTS patients (Fig. 1).

### Discussion

The present study demonstrated lower to normal APC levels in DIC patients in comparison with those observed in the control subjects and non-DIC patients. Taken together with the findings of the previous study [5], which show higher levels of thrombin generation markers, the results indicate that APC levels are insufficient to prevent systemic thrombin generation in DIC patients.

A study, using the same assay kit used in the present study, failed to demonstrate APC-mediated suppression of thrombin generation in ACOTS patients [2]. The other two studies on ACOTS showed the levels of APC to be about 5 to 10 ng/mL (90 to 175 pM) [3, 6]. The estimated circulating APC levels were 40 pM in healthy subjects [7]. Although higher than normal, the levels in these two studies did not reach to a concentration (70–80 ng/mL) sufficient to inhibit thrombin generation [8]. Furthermore, in vitro study confirmed no alterations in coagulation parameters by thromboelastography at APC concentration of 10 ng/mL (175 pM) [8]. These results indicate that normal to mild increases in APC levels are unlikely to suppress thrombin generation in ACOTS.

Liberal transfusion of fresh frozen plasma containing protein C, a source of APC, would theoretically inhibit thrombin formation and aggravate bleeding in ACOTS. However, the results of the present study may suggest that the control of anticoagulant mechanisms, including protein C, APC, and thrombomodulin system, is crucial to prevent a progression of both DIC and ACOTS to inhibit critical bleeding in trauma patients.

### Conclusions

We demonstrated the normal to decreased levels of APC in DIC patients at the early stage of trauma. Contrary to its definition, increased levels of APC were not observed in patients with ACOTS.

## Abbreviations

ACOTS: acute coagulopathy of trauma-shock
APC: activated protein C
DIC: disseminated intravascular coagulation
ISTH: International Society on Thrombosis and Haemostasis
JAAM: Japanese Association for Acute Medicine
